# Astrocytic Acid-Sensing Ion Channel 1a Contributes to the Development of Chronic Epileptogenesis

**DOI:** 10.1038/srep31581

**Published:** 2016-08-16

**Authors:** Feng Yang, Xiaolong Sun, Yinxiu Ding, Hui Ma, Tangpeng Ou Yang, Yue Ma, Dong Wei, Wen Li, Tianle Xu, Wen Jiang

**Affiliations:** 1 Department of Neurology, Xijing Hospital, the Fourth Military Medical University, Xi’an 710032, China; 2 The Key Laboratory of Cerebrocranial Diseases, Ningxia Medical University, Yinchuan 750004, China; 3 Department of Anatomy, Histology and Embryology, Collaborative Innovation Center for Brain Science, Shanghai Jiao Tong University School of Medicine, Shanghai, 200025, China

## Abstract

Unraveling mechanisms underlying epileptogenesis after brain injury is an unmet medical challenge. Although histopathological studies have revealed that reactive astrogliosis and tissue acidosis are prominent features in epileptogenic foci, their roles in epileptogenesis remain unclear. Here, we explored whether astrocytic acid-sensing ion channel-1a (ASIC1a) contributes to the development of chronic epilepsy. High levels of ASIC1a were measured in reactive astrocytes in the hippocampi of patients with temporal lobe epilepsy (TLE) and epileptic mice. Extracellular acidosis caused a significant Ca^2+^ influx in cultured astrocytes, and this influx was sensitive to inhibition by the ASIC1a-specific blocker psalmotoxin 1 (PcTX1). In addition, recombinant adeno-associated virus (rAAV) vectors carrying a GFAP promoter in conjunction with ASIC1a shRNA or cDNA were generated to suppress or restore, respectively, ASIC1a expression in astrocytes. Injection of rAAV-ASIC1a-shRNA into the dentate gyrus of the wide type TLE mouse model resulted in the inhibition of astrocytic ASIC1a expression and a reduction in spontaneous seizures. By contrast, rAAV-ASIC1a-cDNA restored astrocytic ASIC1a expression in an ASIC1a knock-out TLE mouse model and increased the frequency of spontaneous seizures. Taken together, our results reveal that astrocytic ASIC1a may be an attractive new target for the treatment of epilepsy.

Epilepsy, characterized by a predisposition for unpredictable seizures, affects approximately 50 million people worldwide, making it one of the most frequently occurring brain diseases^1^. Despite the current availability of over two dozen antiepileptic drugs (AEDs), about one-third of patients with epilepsy still have poor seizure control and become medically refractory epilepsy^2^. Moreover, the currently available AEDs are primarily intended to suppress epileptic seizures and, therefore, only treat symptoms rather than cure patients of epilepsy by targeting the underlying pathology^2^. Thus, a deeper understanding of the mechanisms involved in the etiology of epilepsy may help identify novel therapeutic targets for its prevention and treatment.

Epileptogenesis, the process by which the normal brain develops epilepsy, involves a number of alterations in molecular, cellular and neuronal networks^1^. Studies on epileptogenic foci removed by surgical resection from human brain tissue have suggested an important role for astrocytes in epileptogenesis^3^. For example, reactive astrogliosis is one of the most common pathological characteristics in the sclerotic hippocampi of patients with mesial temporal lobe epilepsy (TLE)^4^. The abnormal expression and distribution of certain G-protein coupled receptors and voltage gated ion channels on the surface of reactive astrocytes may lead to excessive intracellular Ca^2+^ accumulation that has the potential to then spread to adjacent astrocytes in the form of a “calcium wave”. Simultaneously, this Ca^2+^ accumulation promotes the release of gliotransmitters, including the excitatory transmitters glutamate and ATP, and, thus, contributes to the generation and spread of seizure activity^3^,^5^,^6^. In addition to astrogliosis, chronic inflammation has been observed in epileptic human brain tissue^7^,^8^. Along these lines, recent evidence suggests that inflammation is also an important factor in the initiation of seizures and epileptogenesis^9^,^10^. However, the mechanisms underlying epileptogenesis and the role of inflammation are not yet fully understood.

One important aspect of inflammation is tissue acidosis, where the extracellular pH drops as low as 5.4^11^,^12^,^13^. Low pH activates a unique family of acid-sensing ion channels (ASICs), which are voltage-independent, proton-gated cation-selective channels belonging to the degenerin/epithelial Na^+^ channel (DEG/ENaC) superfamily^14^. While many ASICs are largely Ca^2+^ impermeable, homomeric ASIC1a is Ca^2+^-permeable and highly sensitive to protons, making it a particularly interesting candidate to study in the context of epilepsy^15^,^16^. ASIC1a is widely expressed throughout the central nervous system, especially in the amygdala, hippocampus, cingulate cortex, somatosensory cortex, striatum and periaqueductal gray, and has functional implications in synaptic plasticity, learning and memory^17^,^18^,^19^. Our recent pilot experiment revealed that reactive astrocytes in the sclerotic hippocampi of epileptic mice express ASIC1a at high levels. Therefore, we hypothesized that ASIC1a in reactive astrocytes may contribute to the development of chronic epileptogenesis.

Accordingly, in this study, we first measured the expression of ASIC1a in the sclerotic hippocampi of epileptic mice and TLE patients, as well as in lipopolysaccharide (LPS)-activated astrocytes *in vitro.* Next, the effect of ASIC1a activation on intracellular Ca^2+^ levels in reactive astrocytes was determined *in vitro*. Last, the role of hippocampal astrocytic ASIC1a in the development of chronic epileptogenesis was examined by down-regulating and restoring ASIC1a expression in reactive astrocytes of wild-type mice and ASIC1a knock-out (ASIC1a^−/−^) mice, respectively.

## Results

### High levels of ASIC1a expression in hippocampal reactive astrocytes of epileptic mice and TLE patients

**Figure 1:**
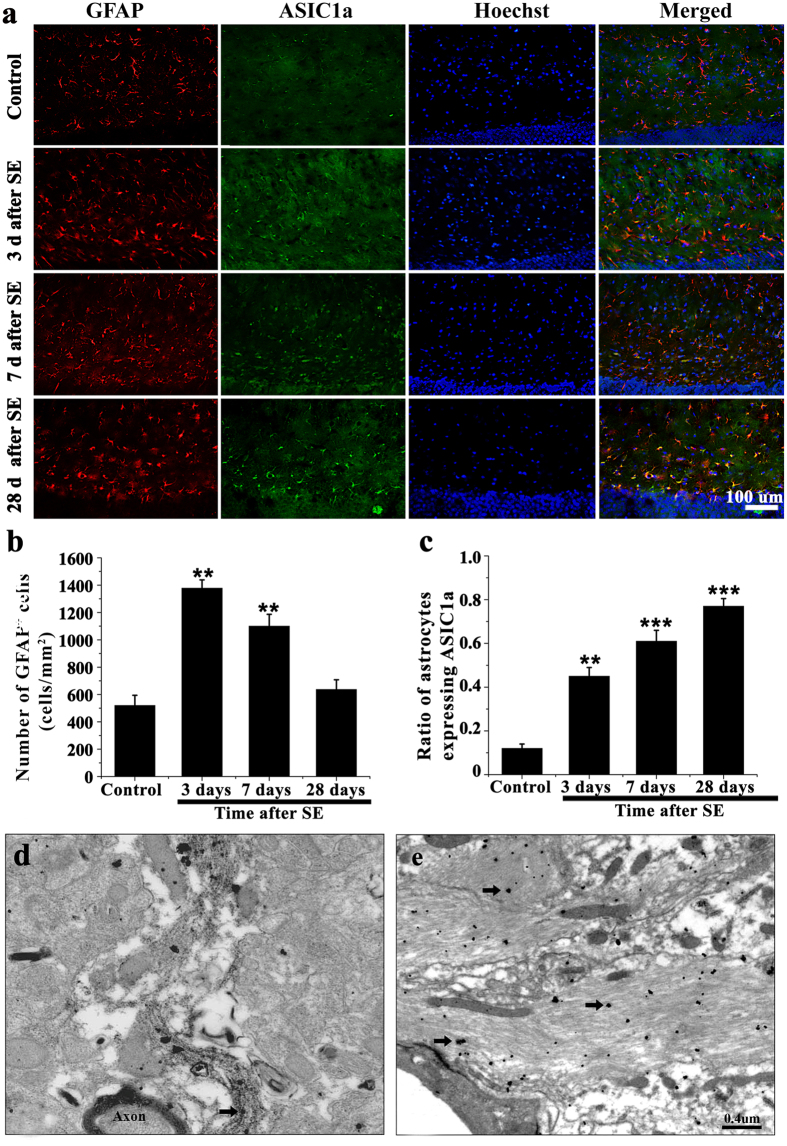
ASIC1a was abundantly expressed in hippocampal astrocytes of epileptic mice. **(a)** GFAP (red), ASIC1a (green), and Hoechst (blue) immunofluorescence staining was performed on control tissue and at 3, 7, and 28 days after SE. **(b)** The number of GFAP positive cells peaked on day 3 after SE. **P < 0.01, ANOVA followed by Dunnett’s post-hoc test vs. control. **(c)** The fraction of astrocytes expressing ASIC1a increased between 3 and 28 days after SE. **P < 0.01, ***P < 0.001; ANOVA followed by Dunnett’s post-hoc test vs. control. **(d,e)** Electron microscopy showed astrocytic ASIC1a (black arrow) in control (**d**) and epileptic (28 days after SE) (**e**) mice.

It has been recently reported that ASIC1a is expressed in astrocytes in normal brain tissue^20^. However, its expression profile in the epileptic brain has not been elucidated. Therefore, we first examined the co-expression of ASIC1a and Glial fibrillary acidic protein (GFAP, a marker of astrocytes) in the mouse hippocampus at 3, 7, and 28 days after SE. In the dentate gyrus, the number of astrocytes at 3 days post-SE was significantly greater than control. On day 7, astrocyte numbers were still elevated compared to control, but decreased slightly compared to day 3. On day 28, astrocyte number declined to control levels (*P* > 0.05) (Fig. 1a,b). However, the ratio of astrocytes expressing ASIC1a increased significantly from day 3 to day 28 (42% and 78%, respectively), and was significantly elevated in all post-SE groups compared to control (Fig. 1c) (P < 0.001). To investigate the precise subcellular localization of ASIC1a in the dentate gyrus, electron microscopy was performed. Very little ASIC1a was expressed on the thin (non-reactive) astrocytic processes in control mice (Fig. 1d). While in epileptic mice (28 days post-SE), ASIC1a was abundant in the hypertrophic cellular processes of reactive astrocytes (Fig. 1e). These data suggest that, in sclerotic hippocampi, ASIC1a expression is significantly up-regulated in reactive astrocytes.

**Figure 2:**
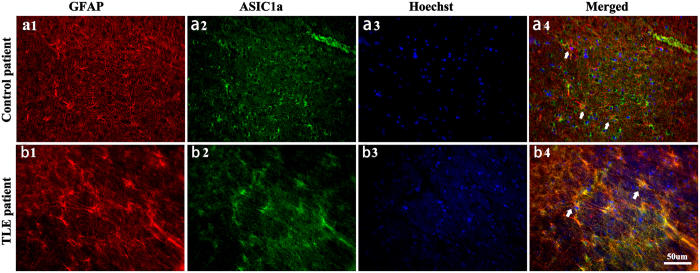
ASIC1a was abundantly expressed in hippocampal astrocytes of TLE patients. **(a1–b4)** GFAP (red), ASIC1a (green), and Hoechst (blue) immunofluorescence staining was performed on TLE patients and control (traumatic brain injury caused by car accidents) patients. **(a1–a4)** There was only a faint expression of ASIC1a on the hippocampal astrocyte in the 4 cases of control patients, with more seeming to be located on neuron-like cells. **(b1–b4)** In all the 4 cases of TLE patients, ASIC1a was abundantly expressed in activated hippocampal astrocytes, which displayed hypertrophy of cell bodies and processes.

To confirm the expression of astrocytic ASIC1a in human brain, we double-labeled GFAP and ASIC1a in the sclerotic hippocampal tissue resected from TLE patients and control (traumatic brain injury caused by car accidents) patients. There was only a faint expression of ASIC1a on the hippocampal astrocytes in the control patients (Fig. 2a1–a4), with most located on neuron-like cells (Fig. 2a2), which was consistent with previous report^20^. While in the TLE patients, ASIC1a was abundantly expressed in hippocampal activated astrocytes, which displayed hypertrophy of cell bodies and processes (Fig. 2b1–b4). Interestingly, ASIC1a was mainly located in membrane and cytoplasm of reactive astrocytes. Because the hippocampal resections were incomplete, hippocampal subfields could not be identified.

### Increased ASIC1a expression in cultured astrocytes

**Figure 3:**
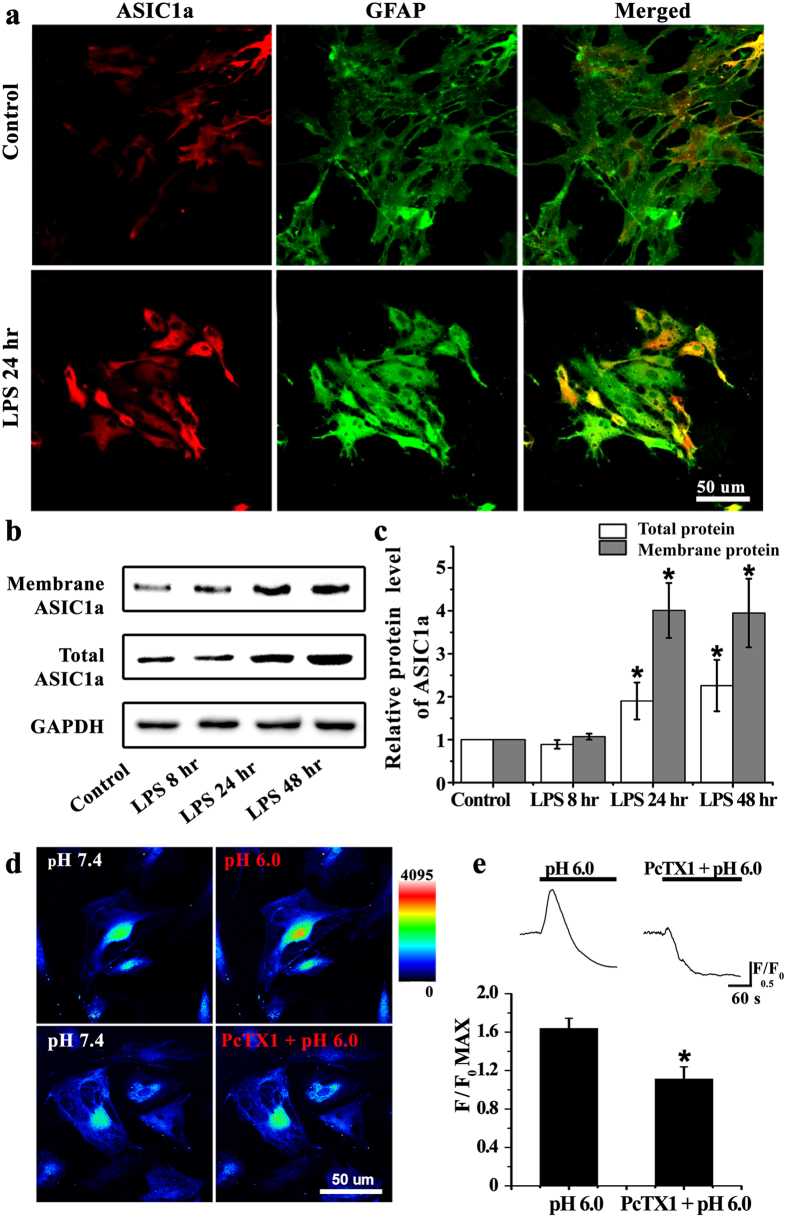
Increased expression of astrocytic ASIC1a mediated Ca^2+^ elevation in LPS-treated astrocytes. **(a)** ASIC1a (red) and GFAP (green) immunofluorescence staining was performed in cultured astrocytes. Compared with the control group, the number of ASIC1a-positive astrocytes markedly increased at 24 hours after LPS treatment. **(b)** Western blots for membrane and total ASIC1a protein in control and LPS-treated astrocytes at 8, 24, and 48 hours after treatment. **(c)** Densitometric quantification of the membrane and total ASIC1a protein expression, normalized to GAPDH. *P < 0.05, ANOVA followed by Dunnett’s post-hoc test vs. control. **(d)** Representative changes of intracellular Ca^2+^ concentration in response to extracellular pH reduction (from 7.4 to 6.0) in the absence and presence of 5 nM PcTX1. Warmer colors indicated higher fluorescence intensity. **(e)** Time course and quantification of fluorescence intensity. Peak fluorescence intensity was normalized to fluorescence intensity at baseline (F/F_0_ max) in the pH 6.0 (n = 24) and PcTX1 + pH 6.0 (n = 21) groups. *P < 0.05, *t* test vs. pH 6.0 group.

To further confirm the upregulation of ASIC1a in reactive astrocytes, primary astrocytes were treated with LPS (2 μg/ml), an inflammatory stimulus, to mimic reactive astrocytes *in vivo*. Immunofluorescence and western blot were performed to detect the ASIC1a expression. Compared with the untreated control group, at 24 hours after LPS pretreatment, astrocytes were hypertrophied and showed thicker ASIC1a and GFAP-positive staining (Fig. 3a). Also, immunoblot analysis showed that, compared to control, total and membrane ASIC1a protein levels were elevated at 24 and 48 (but not 8) hours after LPS treatment (Fig. 3b,c).

### ASIC1a-mediated Ca^2+^ elevation in cultured astrocytes

ASIC1a activation allows high levels of Ca^2+^ influx^21^. We therefore explored whether ASIC1a activation affects intracellular Ca^2+^ levels in reactive astrocytes. Astrocytes were activated by pre-treatment with LPS (2 μg/ml) for 24 hours. Ca^2+^ imaging showed that the intracellular Ca^2+^ concentration increased when the extracellular pH was reduced from 7.4 to 6.0, which could be partially abolished by the ASIC1a-specific antagonist psalmotoxin 1 (PcTX1) (5 nM) (Fig. 3d). The matching traces were plotted in Fig. 3e. Reducing the pH to 6.0 caused a transient increase of normalized fluorescence intensity to 64.0 ± 10.4% above baseline (F/F_0_ max, n = 24, Fig. 3e) with a long lasting decrease. Co-application of PcTx1 attenuated this increase to 11.3 ± 12.5% above baseline (F/F_0_ max, n = 21, Fig. 3e), suggesting that PcTx1 significantly suppressed the acid-induced Ca^2+^ influx. Therefore, the acid-induced elevation of astrocytic Ca^2+^ is mediated, at least in part, by ASIC1a channels.

### Efficiency of viral transduction

**Figure 4:**
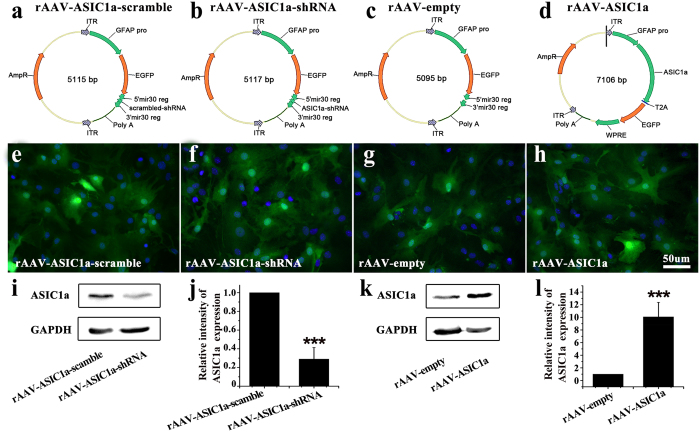
rAAV plasmids and virally induced ASIC1a expression in cultured astrocytes. **(a–d)** Schematic of the four rAAV plasmids used: rAAV-ASIC1a-scramble virus carrying a GFAP-EGFP-ASIC1a-scrambled-shRNA vector, rAAV-ASIC1a virus carrying a GFAP-EGFP-ASIC1a-shRNA vector, rAAV-empty virus carrying a GFAP-EGFP vector, and rAAV-ASIC1a virus carrying GFAP-ASIC1a-EGFP vector. **(e–h)** EGFP and Hoechst fluorescence in astrocyte cultures at 72 hours after virus transduction. **(i,k)** Immunoblots for total ASIC1a protein after viral transduction. **(j,l)** Densitometric quantification of ASIC1a, normalized to GAPDH. ***P < 0.001, *t* test vs. scramble or empty vector.

The efficiency of viral ASIC1a transgene expression was first examined using cultured astrocytes from WT mice at 72 hours after virus transduction. Astrocytes were imaged for reporter Enhanced green fluorescent protein (EGFP) fluorescence. As shown in Fig. 4e–h, the ratios of EGFP-positive astrocytes were 96.6 ± 1.2% in rAAV-ASIC1a-scramble group, 94.5 ± 1.5% in rAAV-ASIC1a-shRNA group, 95.8 ± 1.8% in rAAV-empty group, and 93.6 ± 1.1% in rAAV-ASIC1a group, respectively. There were no differences between groups (data not shown). The efficiency of viral transduction was then verified by western blot. rAAV-ASIC1a-shRNA markedly reduced the expression of ASIC1a compared to rAAV-ASIC1a-scramble (Fig. 4i,j). ASIC1a expression was significantly higher in rAAV-ASIC1a than in rAAV-empty-infected cells (Fig. 4k,l).

**Figure 5:**
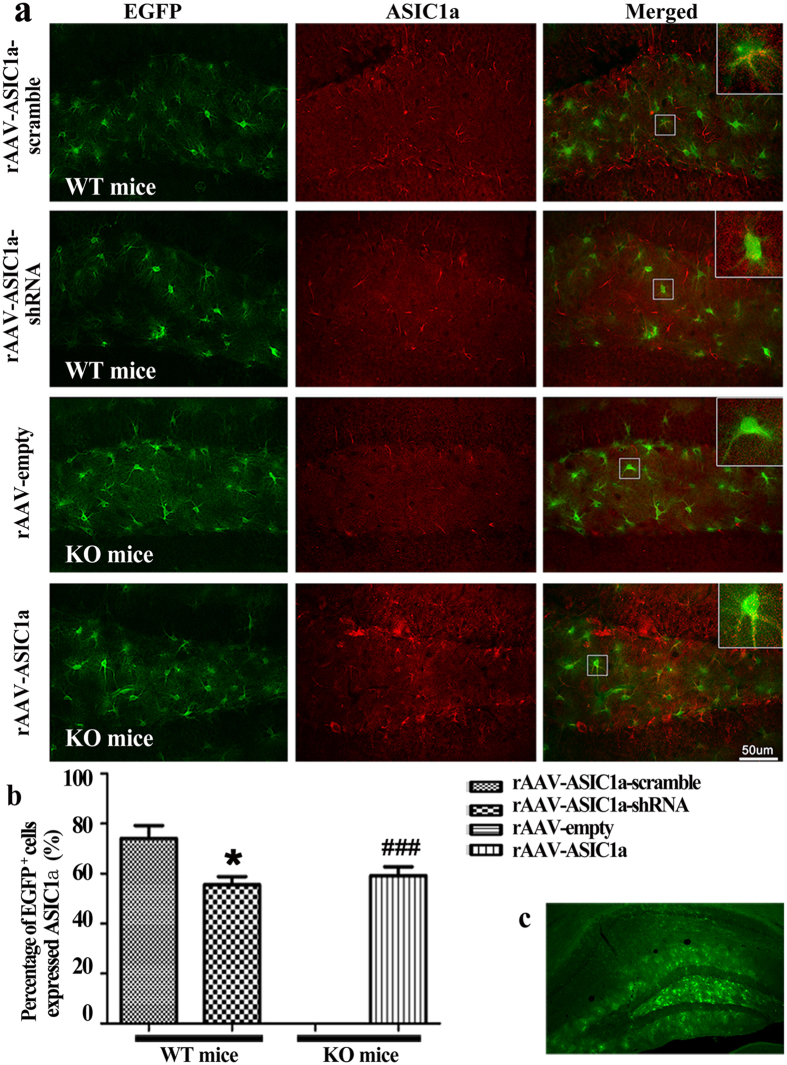
The efficiency of viral vector-induced ASIC1a expression at 5 weeks after SE. **(a)** EGFP and ASIC1a immunofluorescence staining in each group. **(b)** Percent of EGFP-positive cells expressing ASIC1a in each group. All data were shown as mean ± SEM. WT: n = 7, KO: n = 6 mice per group. *P < 0.05, *t* test vs. rAAV-ASIC1a-scramble; ^###^*P* < 0.001, *t* test vs rAAV-empty. **(c)** Low magnification view showing the region of virus transduction in the dentate gyrus.

We then examined viral expression in injected hippocampi of WT and KO mice. AAV-mediated gene expression is reported to peak at 3–4 weeks *in vivo*^22^. Therefore, we examined ASIC1a expression at 4 weeks after virus injection (5 weeks after SE). To minimize viral injection lesions, a single injection point in each dentate gyrus was used. Low magnification images of the hippocampus (Fig. 5c) showed EGFP fluorescence around projection areas, covering over almost all the dentate gyrus. EGFP fluorescence was mainly localized to the polymorph and molecular layers. Weak EGFP fluorescence extended into CA1. Quantification of the co-expression of ASIC1a and EGFP showed that in WT mice, rAAV-ASIC1a-shRNA significantly reduced EGFP and ASIC1a co-expression compared to rAAV-ASIC1a-scramble (57.8 ± 2.6% and 76.4 ± 3.2%, respectively); In KO mice, rAAV-ASIC1a rescued ASIC1a expression in 61.1 ± 1.7% of EGFP positive cells; ASIC1a was absent in EGFP-positive cells from KO mice injected with the rAAV-empty vector (Fig. 5a,b). These results indicated that our viral vectors can efficiently transduce widespread ASIC1a transgene expression in astrocytes *in vivo* and *in vitro*.

### The role of astrocytic ASIC1a in chronic epileptogenesis

**Figure 6:**
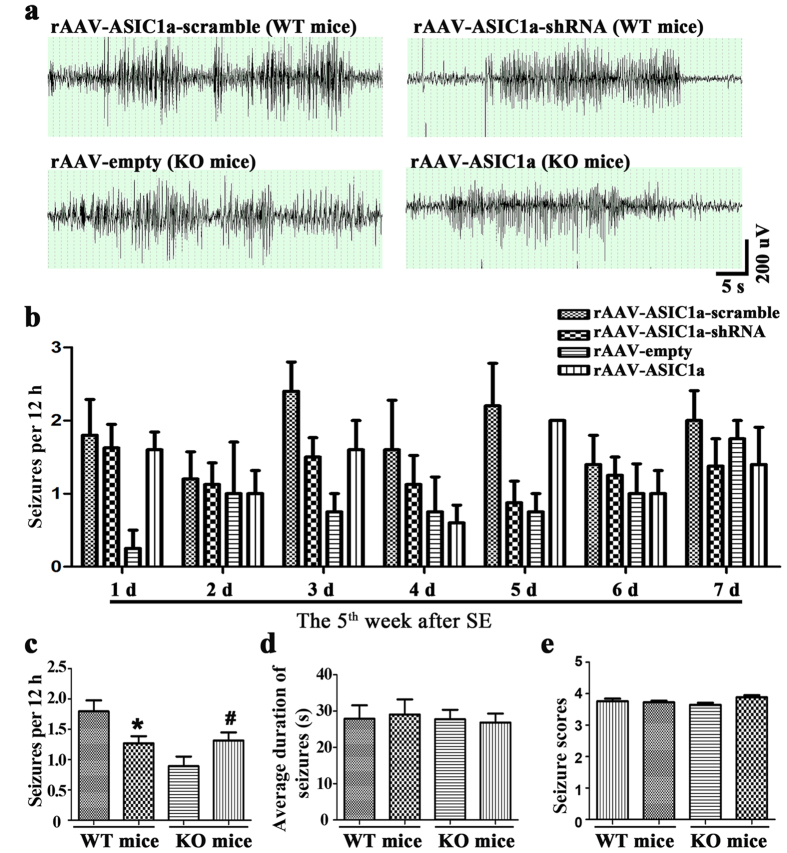
Effects of rAAV vectors on spontaneous seizures in mice during chronic epileptogenesis. **(a)** Ictal EEG signals in each group. **(b)** Spontaneous seizure frequency during the 5^th^ week after SE. **(c–e)** Statistical analysis of spontaneous seizure frequency, total time in seizures, and seizure scores. *P < 0.05, *t* test vs. rAAV ASIC1a-scramble. ^#^*P* < 0.05, *t* test vs. rAAV-empty. Bars represent mean ± SEM. WT: n = 7, KO: n = 6 mice/group.

Lastly, we tried to examine the effects of manipulating hippocampal astrocytic ASIC1a on chronic epileptogenesis. We down-regulated and restored ASIC1a expression in astrocytes of WT and KO mice, respectively, and examined spontaneous seizures during the 5^th^ week after SE. Every spontaneous seizure was carefully confirmed by behavioral features associated with ictal events in EEG recording. Representative ictal EEG recordings of each group were shown in Fig. 6a.

In WT mice, video-EEG analysis showed that seizure frequency in the rAAV-ASIC1a-shRNA group (1.27 ± 0.3 seizures/12 h, n = 6) was lower than the rAAV-ASIC1a-scramble group (1.79 ± 0.4 seizures/12 h, n = 6) (*P* < 0.05). In KO mice, seizure frequency in the rAAV-ASIC1a group (1.31 ± 0.3 seizures/12 h, n = 7) was higher than the rAAV-empty group (0.89 ± 0.3 seizures/12 h, n = 7) (*P* < 0.05) (Fig. 6b,c). No differences in seizure score or average duration of spontaneous seizures were observed between groups (*P* > 0.05) (Fig. 6d,e). Taken together, these results suggested that down-regulation of astrocytic ASIC1a expression in WT mice decreased spontaneous seizures frequency in chronic period of epileptogenesis, while restored the expression of astrocytic ASIC1a in KO mice had the opposite effect.

## Discussion

In this study, we uncovered the following four findings. First, high ASIC1a levels are expressed on reactive astrocytes in the hippocampi of both TLE patients and epileptic mice, as well as in LPS-induced reactive astrocytes *in vitro*. Second, activation of ASIC1a results in a significant increase of intracellular Ca^2+^ levels in reactive astrocytes. Third, down-regulation of astrocytic ASIC1a in the epileptic hippocampi of WT mice inhibits spontaneous seizures. Fourth, restoration of astrocytic ASIC1a expression in the epileptic hippocampi of ASIC1a KO mice increases the frequency of spontaneous seizures. These results provide the first direct evidence that astrocytic ASIC1a is an important contributing factor in the development of epileptogenesis.

Astrocytes, the most abundant glial cell type in the mammalian brain, play essential roles in brain pathophysiology through modulation of synaptic transmission and regulation of ion homeostasis and blood–brain barrier integrity^23^. These cells respond to a range of brain insults through a complex process referred to as reactive astrogliosis. This process involves both morphological and functional changes, including hypertrophy, increased proliferation, and up-regulation of intermediate filaments, such as GFAP^24^. In this study, significant reactive astrogliosis was discovered in the hippocampus during epileptogenesis induced by pilocarpine, which was consistent with our previous study^25^. Reactive astrocytes may contribute to epileptogenesis by losing normal or gaining abnormal astrocytic functions^26^. For example, Eid T *et al*. noted a reduction in the expression of water channel aquaporin 4 and dystrophin on reactive astrocytes in the sclerosis hippocampus of TLE patients. This may result in a perturbed water flux through astrocytes, which can lead to impaired buffering of K^+^ and an increased propensity for seizures^27^. In this work, we provide the first report of high levels of ASIC1a on reactive astrocytes in the sclerosis hippocampus of epileptic mice and TLE patients.

ASIC1a is a member of a novel family of proton-gated amiloride-sensitive cation channels and is expressed primarily in Ca^2+^ and Na^+^ permeable neurons^14^. Activation of these channels results in intracellular Ca^2+^ accumulation, which plays an important role in neurological disorders, such as brain ischemia, multiple sclerosis and spinal cord injury^14^. Recently, Huang C *et al*. found ASIC1 was expressed in cultured and *in situ* astrocytes. Specifically, ASICs were mainly expressed in the nucleus, but not in the membrane/cytoplasm, of astrocytes under physiological condition^20^. In this study, high levels of ASIC1a were found in the membrane/cytoplasm of reactive astrocytes under pathological conditions, suggesting possible trafficking of ASICs in astrocytes in response to certain stimuli. *In vitro* experiments further indicate that astrocytic ASIC1a can sense extracellular acidosis and mediate Ca^2+^ influx, confirming that ASIC1a in the membrane of astrocytes is functional. It is interesting to observe a long-lasting decline of intracellular Ca^2+^ following the transient increase when lowering extracellular pH from 7.4 to 6.0. This phenomenon is likely to be associated with 1) gap junctions between astrocytes, which cause the intercellular propagation of calcium wave, and 2) our experimental conditions, including lifetime of calcium indicators, environment of the laboratory, cell’s state, and so on.

Astrocytic Ca^2+^ signaling plays a pivotal role in communication between astrocytes and neurons^28^. Several lines of evidence indicate that activation of Ca^2+^ signals in astrocytes induces the subsequent release of gliotransmitters, such as glutamate, D-serine, and ATP, which may amplify the propensity for epileptic seizures^6^. In this study, it was found that ASIC1a activation in reactive astrocytes resulted in a significant elevation in cytosolic Ca^2+^ levels. Study *in vivo* revealed that inhibiting astrocytic ASIC1a expression in the sclerotic hippocampus using a rAAV carrying ASIC1a shRNA reduced the frequency of spontaneous seizures. Furthermore, restoring astrocytic ASIC1a expression in the sclerotic hippocampus of ASIC1a knock-out mice using a rAAV carrying ASIC1a cDNA displayed the opposite phenotype. These results suggest that astrocytic ASIC1a activation increases the excitability of the hippocampal local circuit and contributes to the development of epileptogenesis. However, the detailed mechanisms linking astrocytic ASIC1a activation and neuronal hyperexcitability require further delineation.

In the literature, the exact roles of ASICs in seizure generation, propagation and termination have been controversial^29^. A number of *in vivo* studies have found amiloride, a commonly used non-selective ASIC blocker, has an anticonvulsant effect in pilocarpine, pentylenetetrazole and febrile seizure models^30^,^31^,^32^,^33^. In addition, some *in vitro* studies revealed that ASIC1 activation might be proconvulsant in a cell culture model of epilepsy and in hippocampal slices^29^. However, Ziemann and colleagues found that ASIC1a activation was involved in the termination of epileptic seizure activity because ASIC1a expression levels were higher in GABAergic interneurons than in excitatory neurons^34^. From this it was concluded that ASIC1a activation limited seizures by increasing inhibitory tone. In this paper, we focused on the development of chronic epileptogenesis, during which period reactive astrogliosis and significant loss of GABAergic interneurons predominate^35^,^36^,^37^. Expression of ASIC1a channels was abundant in reactive astrocytes, and activation of these channels led to the elevation of intracellular Ca^2+^ concentrations, and, thus, contributed to the development of chronic epileptogenesis. In fact, ASIC1a channels in both neurons and astrocytes could be activated simultaneously when acidosis occurs in the same tissue. The influence of ASIC1a on the neural network depends on the integrated effects of ASIC1a on different neuronal cells. So, due to the imbalance of ASIC1a expressions on reactive astrocytes and GABAergic interneurons, the effect of ASIC1a on neural network in chronic epileptogenesis may depend on hyper-excitability mediated by reactive astrocytes. Therefore, our study is not in conflict with the results published by Ziemann and colleagues. Rather, this work contributes to our understanding of ASIC1a functions in epilepsy.

ASICs belong to the DEG/ENaC superfamily^17^. To date, there are seven subunits of ASICs (1a, 1b1, 1b2, 2a, 2b, 3, and 4) that have been identified, which are encoded by four genes^11^. Besides ASIC1a, several other ASIC subunits have been reported to have a role in epileptic seizures. Wu H *et al*. found that pilocarpine-induced seizures were associated with significant up-regulation of ASIC2a in the piriform cortex, and that inhibition of ASICs, particularly ASIC2a, suppressed seizures originating in this region^38^. Cao Q *et al*. reported that ASIC3 expression was elevated in TLE patient and epileptic rat brains, and co-localized with astrocyte marker GFAP and inhibitory GABAergic interneuron marker GAD67. Intracerebroventricular injection of the ASIC3 inhibitor APETx2 shortened the latency to seizure and increased the incidence of generalized tonic clonic seizure in experimental epilepsy models, suggesting that elevated levels of ASIC3 may be antiepileptic^39^. However, studies remain to be conducted on whether reactive astrocytes in the sclerosis hippocampus abundantly express ASIC2a and/or ASIC3 and what is these channels’ function in this context.

To elucidate the role of astrocytic ASICs in brain function, it is necessary to manipulate their expression *in situ* where neuronal/glial interactions remain intact. Such manipulations include molecular genetics, imaging, and use of transgenic animals. However, these approaches are technically challenging, time consuming, and have variables that are difficult to control^40^. Gene transfer is a valuable method by which to target astrocytes and explore their functions in experimental disease models. In particular, viral-mediated gene transfer is a rapid, flexible and cost-effective *in vivo* technique that can be used to investigate the function of genes of interest in adult brains^41^. The rAAV vector in particular is commonly used both *in vivo* and *in vitro* due to its high efficiency, stability, minimal immunogenicity, and broad host and cell range^42^. In this study, we employed high-titer AAV2 vectors carrying a 682 bp GFAP promoter, an efficient and specific method to regulate gene expression in astrocytes^43^.

Although the present study has yielded some important findings concerning ASIC1a and its role in epileptogenesis, some limitations should be noted. First, the rAAV vectors used to manipulate astrocytic ASIC1a expression were only transduced into the dentate gyrus of the sclerotic hippocampi, which might be insufficient to cover all of the epileptogenic regions in the pilocarpine TLE model^44^. This could, at least in part, be the reason that inhibition of astrocytic ASIC1a reduced, but failed to completely abrogate, spontaneous seizures. Second, continuous video-EEG monitoring and behavioral observation were performed for a maximum of seven days. Although statistically significant differences were found in the frequency of spontaneous seizures among the different experimental groups within this timeframe, there is a cyclicity of spontaneous recurrent seizures in the pilocarpine TLE model^45^ that warrants longer behavioral and EEG monitoring in the future studies. Third, further investigations are needed to elucidate the deeper *in vivo* underlying mechanism of epileptogenesis induced by astrocytic ASIC1a activation.

**Figure 7:**
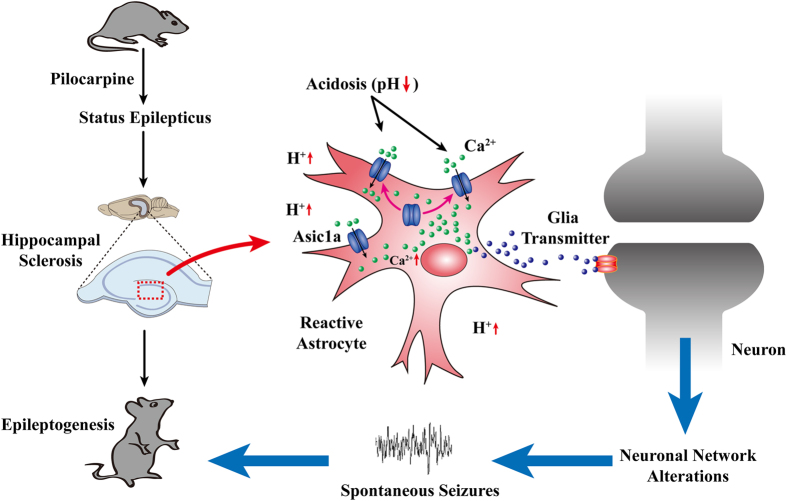
Proposed role of astrocytic ASIC1a in chronic epileptogenesis. Acute brain injury such as status epilepticus can cause hippocampal sclerosis. Reactive astrogliosis and tissue acidosis are the prominent features in sclerotic hippocampi. Reactive astrocytes express a high level of ASIC1a, which can be activated by local extracellular low pH. This would lead to excessive Ca^2+^ influx in astrocytes and release of gliotransmitters, thus resulting in epileptiform activities and the development of chronic epileptogenesis.

In conclusion, our study revealed that astrocytic ASIC1a plays a vital role in epileptogenesis (Fig. 7) and has the potential to serve as a novel target for the treatment of epilepsy.

## Materials and Methods

### Animals

Wild-type (WT) and ASIC1a knock-out (KO) mice weighing 20–25 g were used. Mice were maintained on a congenic C57BL/6 background. Animals were housed at 24–25 °C and 50–60% humidity on a 12-hour light/dark cycle (lights on at 8:00 A.M.). Food and water were provided *ad libitum*. All experimental procedures were approved by the Fourth Military Medical University Animal Care Committee in strict accordance with the guidelines established by the US National Institutes of Health.

### rAAV-vector production

We specifically targeted astrocytes using a recombined AAV2 vector carrying a 682-bp mouse GFAP promoter^43^. The construction and production of vectors were performed as our previous study^43^,^46^,^47^. The shRNA targeting mouse ASIC1a was designed as follows: 5′-TCGA**CTATGCCTATGAGGTCATTAA***CTCGAG***AATGACCTCATAGGCATAGTC**TTTTTT-3′ (bold: shRNA-sense and -antisense strands, respectively; italics: hairpin turn). The control (scrambled) shRNA sequence was: 5′-TCGA**TTCTCCGAACGTGTCACGTTT***CAAGAG***AACGTGAC11ACGTTCGGAGAA**TTTTTTG-3′. ASIC1a-shRNA, ASIC1a-scramble or ASIC1a-cDNA was subcloned into an expression cassette consisting of the mouse GFAP promoter. EGFP was used as a reporter gene. The recombinant plasmid was identified by DNA sequencing (Fig. 4a–d).

### Pilocarpine-induced SE

To induce status epilepticus (SE), a single dose of pilocarpine (130 mg/kg, i.p., Sigma) was given at 18 hours after lithium chloride (350 mg/kg, i.p., Sigma) administration. After 60 minutes SE, seizures were terminated with diazepam (30 mg/kg, i.p.). Subjects were closely observed during SE and given supportive care (food soaked in a 5% glucose solution for 3 days) until normal eating and drinking behavior resumed.

This study consisted of two experiments with specific aims. Experiment 1 aimed to examine the expression pattern of astrocytic ASIC1a in epileptogenesis. WT mice were divided into two groups. Group 1 (n = 5) comprised the control group, which did not undergo SE. SE was induced in group 2 (n = 24) animals. Of the 24 mice injected with LiCl-pilocarpine, 22 mice developed SE, and 4 mice died during or shortly after SE. The remaining 18 mice were perfused for histology 3, 7, and 28 days after SE (n = 6 for each time point), respectively.

**Figure 8:**
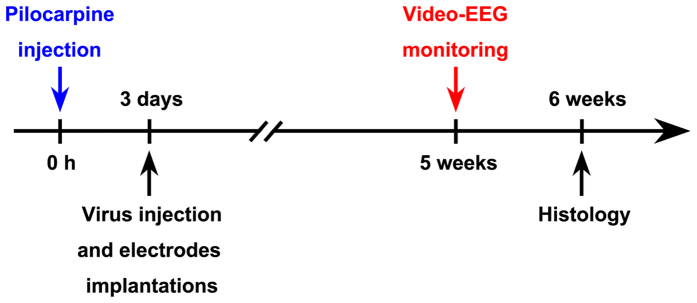
The flow chart of pilocarpine injection, virus injection and electrodes implantations, video-EEG monitoring and histology for Experiment 2 in the Materials and Methods.

Experiment 2 aimed to investigate the role of astrocytic ASIC1a in epileptogenesis. 20 WT and 20 KO mice received LiCl-pilocarpine to induce SE. 18 WT and 17 KO mice developed SE. Of these, 4 WT and 5 KO mice died during or shortly after SE. The flowchart of experimental 2 procedure was shown as in Fig. 8.

### rAAV-vector injection and EEG electrode implantation

Three days after SE, WT mice in experiment 2 were randomly assigned to 2 groups, receiving bilateral hippocampal injections of either rAAV-ASIC1a-scramble or rAAV-ASIC1a-shRNA (n = 7/group). KO mice in experiment 2 were randomly assigned to 2 groups, receiving bilateral hippocampal injections of rAAV-empty or rAAV-ASIC1a (n = 6/group). Animals were anesthetized with chloral hydrate (400 mg/kg, i.p.) and secured in a stereotaxic frame. rAAV-vectors (2 μL, 1.09 × 10^12^–1.15 × 10^12^ particles/mL) were injected bilaterally into the dentate gyrus (AP: −2.00 mm, LM: 1.40 mm, DV: 2.00 mm).

At the same time, polyimide-insulated silver wire monopolar depth electrodes (outside diameter 0.14 mm) were bilaterally implanted into CA1 (AP: −2.06 mm, LM: 1.60 mm, DV: 1.40 mm). A reference electrode was placed into the right frontal lobe (AP: 1.20 mm, LM: 1.80 mm, DV: 0.50 mm). A ground electrode was placed in the prefrontal bone^48^,^49^. Each electrode was soldered to a single socket contact and attached to a plug using dental cement.

All experimental animals survived during rAAV injection period and EEG electrode implantation period. Then, continuous video-EEG monitoring was performed to record the change of EEG and behavior seizures on the 5^th^ week after SE.

### Continuous video-EEG monitoring and behavioral analysis

During the 5^th^ week post-SE, animals were continuously monitored for 12 hours/day by video-EEG. EEG activity from hippocampal electrodes was amplified, filtered (high pass: 0.1 Hz; low pass: 45 Hz), and recorded (100 Hz sampling rate) with an EEG Amplifier System (SOLAR3000N, China). EEG seizures were defined as the paroxysmal activity of high frequency (>5 Hz) and amplitude (3-fold greater than baseline) lasting more than 20 s^50^. Spontaneous behavioral seizures corresponded to paroxysmal alterations in the EEG recording^51^. Seizure severity was scored on the Racine scale based on the video recording^52^.

Video-EEG recordings were examined and analyzed by two independent, blinded investigators. In the event of different evaluations, investigators reviewed the recordings together to reach a consensus.

### Patient evaluation and tissue preparation

Four refractory TLE patients (2 men, 2 women, mean age 22.75 ± 8.85 years, mean TLE history 14 ± 4.32 years) with unilateral hippocampal sclerosis identified by MRI were included in this study. The diagnosis was made by previously reported clinical and electrographic characteristics (Commission on Classification and Terminology of the International League Against Epilepsy, 1989). Four control patients, 3 men, 1 woman, mean age 32.2 ± 8.6 years, with traumatic brain injury caused by car accidents, were included in this study. All patients had undergone decompression surgery within six hours post-traumatic brain injury, and then parts of hippocampi were carefully isolated from tissues resected. Surgically resected sclerotic hippocampal tissue was dissected and fixed in 4% paraformaldehyde overnight at 4 °C. Specimens were embedded in paraffin after gradient alcohol dehydration. Paraffin-embedded brains were cut into 5 μm sections for immunofluorescence staining following standard laboratory procedures. The use of human tissue samples were approved by the Ethics Committee of Xijing Hospital. The patients provided written informed consent.

### Primary culture of cortical astrocytes

The cerebral cortex was isolated from one-day-old C57BL/6J pups. Cells were dissociated by pipetting and trypsinization (0.125% trypsin with 0.02% EDTA, 15 minutes). Trypsinization was arrested with 1 mL trypsin inhibitor (0.125 mg/mL; Sigma). The suspension was then centrifuged (800 rpm, 5 minutes) and cells were resuspended in DMEM/F-12 (Invitrogen) supplemented with 15% fetal bovine serum (FBS). Cultured cells were maintained at 37 °C in humidified 95% oxygen/5% CO_2_. At approximately 14 days *in vitro*, primary astrocytes were shaken at 280 rpm overnight to remove non-astrocytic cells. Purified astrocytes were used for experiments after two passages.

### Immunofluorescence staining

Immunofluorescence staining was performed as previously described 46. Briefly, frozen sections or cells were incubated with anti-ASIC1a goat polyclonal (1:50, Santa Cruz) and anti-GFAP mouse monoclonal (1:3000, Sigma) antibodies overnight at 4 °C, followed by the appropriate secondary antibodies (1:500; Cell Signaling) for 2 hours at room temperature. Nuclei were counterstained with Hoechst 33258 for 5 minutes at room temperature. Frozen sections or cells were imaged on a confocal microscope (FV1000, Olympus, Japan). ASIC1a/GFAP immunofluorescence analysis was performed as described previously^46^,^53^. Five coronal hippocampal sections were selected from each mouse. Total numbers of GFAP^+^ or ASIC1a^+^ cells were counted in a 5 × 10^4^ μm^2^ area.

### Immuno-electron microscopy

Brain tissue immunogold staining was performed as described previously^54^. Briefly, after fixing, dehydrating, saturating and embedding, coronal sections (70 nm) were incubated with anti-ASIC1a goat polyclonal (1:50, Santa Cruz) or anti-GFAP mouse monoclonal (1:3000, Sigma) antibodies overnight at 4 °C. Sections were then incubated with colloidal gold (5 nm or 10 nm)-conjugated secondary antibodies (rabbit anti-goat and anti-mouse IgG (Amersham)) for 2 hours at room temperature. Sections were rinsed with distilled water, dyed with 1% PVA containing 0.1% uranyl acetate, dried, then imaged using a Transmission Electron Microscope (Hitachi7500, Japan).

### Western Blot analysis

Briefly, tissue or cells were lysed in RIPA buffer containing 2% protease inhibitor (Gibico). Lysates were separated on SDS-polyacrylamide gels and transferred to PVDF membranes (Millipore). Membranes were incubated with primary antibodies (goat anti-ASIC1a 1:200, Santa Cruz) and HRP-conjugated secondary antibodies (mouse anti-goat, 1:3000, Millipore), then developed with ECL (Millipore). Band intensities were quantified using Image-ProPlus 6.0 software. ASIC1a band intensity was normalized to GAPDH as a loading control.

### Ca^2+^ imaging

Ca^2+^ imaging was performed as described previously^54^,^55^. Briefly, cultured astrocytes were pretreated with LPS (2 μg/ml) for 24 hours, then incubated with 5 μM fura-4 (Sigma-Aldrich) in PBS for 30 minutes at 37 °C. Fura-4 fluorescence was excited using a 494 nm laser line and emitted using a wavelength of 516 nm by an inverted microscope (Olympus, FV1000). Digitized images were acquired and analyzed in a PC controlled by FV10-ASW 3.1. Ratio images were analyzed by averaging pixel ratio values in circumscribed regions of cells in the field of view. The values were exported to Origin 8.0 for additional analysis.

### Statistical analysis

All data are expressed as mean ± SEM. Statistical comparisons were performed using Student’s *t* tests or one-way ANOVAs with Dunnett’s post-hoc test, as indicated. All statistical analyses were performed using SPSS v16.0. *P* values below 0.05 were considered statistically significant.

## Additional Information

**How to cite this article**: Yang, F. *et al*. Astrocytic Acid-Sensing Ion Channel 1a Contributes to the Development of Chronic Epileptogenesis. *Sci. Rep.* **6**, 31581; doi: 10.1038/srep31581 (2016).
