# Corrigendum: Astrocytic Acid-Sensing Ion Channel 1a Contributes to the Development of Chronic Epileptogenesis

**DOI:** 10.1038/srep38593

**Published:** 2016-12-06

**Authors:** Feng Yang, Xiaolong Sun, Yinxiu Ding, Hui Ma, Tangpeng Ou Yang, Yue Ma, Dong Wei, Wen Li, Tianle Xu, Wen Jiang

Scientific Reports 6: Article number: 3158110.1038/srep31581; published online: 08 16 2016; updated: 12 06 2016

The Acknowledgements section in this Article is incomplete.

“This work was supported by grants from The National Science Foundation of China (No. 81271432 and 81571262). The funders had no role in study design, data collection and analysis, decision to publish, or preparation of the manuscript.”

should read:

“This work was supported by grants from The National Science Foundation of China (No. 81271432 and 81571262). The funders had no role in study design, data collection and analysis, decision to publish, or preparation of the manuscript. We thank Dr. Michael J. Welsh for kindly providing the ASIC1a knockout mouse used in the current study.”

